# P29 Transforming antimicrobial stewardship practice: a real-time dashboard revolution for combating antimicrobial resistance

**DOI:** 10.1093/jacamr/dlaf230.036

**Published:** 2025-12-04

**Authors:** Rasha Abdelsalam Elshenawy, Nkiruka Umaru, Zoe Aslanpour

**Affiliations:** Department of Medicine, School of Health, Medicine and Life Sciences, University of Hertfordshire, UK; Department of Medicine, School of Health, Medicine and Life Sciences, University of Hertfordshire, UK; Department of Medicine, School of Health, Medicine and Life Sciences, University of Hertfordshire, UK

## Abstract

**Background:**

Antimicrobial resistance (AMR) caused 1.27 million deaths in 2019 and could reach 10 million annually by 2050.^1^ In the UK, 67 000 AMR infections in 2023 led to 2200 deaths.^2^ Despite recognition of antimicrobial stewardship (AMS), monitoring remains fragmented, retrospective and disconnected from real-time decision-making.^3^ Building on our research and parliamentary advocacy embedding AMS into UK healthcare, this study addresses the critical policy–practice gap through innovative digital technology [4], advancing stewardship implementation and improving patient safety outcomes.

**Objectives:**

This study aims to develop and validate a real-time antimicrobial stewardship dashboard that integrates prescribing, microbiology and outcome data. Aligned with the Start Smart, Then Focus framework, it enables proactive decision support and evidence-based quality improvement at the point of care.

**Methods:**

Our evidence-based process synthesized findings from three UK NHS studies (2019–2024). A systematic review of 8763 articles revealed key monitoring gaps.^1^ Retrospective analysis of 640 patient records highlighted prescribing patterns and intervention opportunities.^2^ A survey of 240 healthcare professionals identified barriers to stewardship.^3^ Dashboard development used a four-phase methodology: gap analysis, user-centred design, Excel-based technical development for accessibility and evidence-based content aligned with Start Smart, Then Focus. Validation involved technical testing and expert review.

**Results:**

The dashboard employs dual-phase monitoring, capturing empirical and pathogen-directed therapy. The Start Smart module shows that community-acquired pneumonia accounts for 40% of respiratory admissions, with only 47% of reviews completed within 48–72 h. Then, the Focus module tracks isolates, decision timelines and discharge management. Interactive filters allow hospital-to-ward analysis. Validation confirmed accuracy and clinical approval. Its Excel-based design ensures affordability while detecting inappropriate broad-spectrum use beyond 72 h and enabling real-time tracking of de-escalation opportunities, supporting evidence-based stewardship practice.

**Conclusions:**

This dashboard marks the next stage in our antimicrobial stewardship research, progressing from policy advocacy to practical digital solutions that transform clinical practice. It shifts stewardship from retrospective auditing to proactive decision support, providing ward-level monitoring that enables targeted education and systematic quality improvement. By making stewardship visible, measurable and actionable, it bolsters global efforts against antimicrobial resistance. Demonstrated feasibility through accessible technology offers a scalable model for healthcare systems worldwide, with the potential to save countless lives.
